# Antenatal breast expression in women with diabetes: outcomes from a retrospective cohort study

**DOI:** 10.1186/1746-4358-7-18

**Published:** 2012-12-01

**Authors:** Hora Soltani, Alexandra MS Scott

**Affiliations:** 1 Sheffield Hallam University, 32 Collegiate Crescent, Sheffield, UK

**Keywords:** Diabetes, Antenatal, Breast milk expression, Retrospective, Gestational age, Cohort, Gestation

## Abstract

**Background:**

Women with diabetes are sometimes advised to express breast milk antenatally to prepare for breastfeeding and to store colostrum for infant feeding in preventing or treating hypoglycaemia after the birth. The acceptability, risks and benefits of this practice have not been evaluated. This was aimed to investigate the pattern of antenatal breast expression uptake and its relationship with birth outcomes in women with diabetes.

**Methods:**

This was part of a two year retrospective cohort study of pregnant women with diabetes (type 1, 2 and gestational diabetes) who gave birth during 2001–2003 in Derby Hospitals NHS Foundation Trust (n = 94). The information on the practice of antenatal breastfeeding expression and birth outcomes was collected via self-administered questionnaires and by examining maternity records.

**Results:**

Thirty-seven percent of women (35/94) recalled that they were advised to express antenatally and 17% did (16/94). The mean gestational age at birth for women who hand-expressed was lower than that for those who did not (mean difference (MD) (95% confidence intervals (CI)): -1.2 (−2.4 to 0.04), p = 0.06). A higher proportion of babies from the antenatal expression group were admitted to special care baby units (SCBU) (MD (95% CI): 21% (−3.9 to 46.3).

**Conclusions:**

Less than half the women who stated that they were advised to express, did so. There seems to be an indication that antenatal breast milk expression and lower gestational age at birth are associated. The trend of a higher rate of SCBU admission for babies from the breast milk expression group compared to those who did not express antenatally is of concern. An appropriately-powered randomised controlled trial is needed to determine the safety of this practice and its acceptability to women and health professionals before it can be recommended for implementation in practice.

## Background

There are benefits of breastfeeding and avoiding the use of formula milk for all mothers and babies, with evidence to suggest that breastfeeding has additional benefits for women with diabetes and their children [1]. Women with diabetes often have more complications and medical interventions during pregnancy than women who do not have diabetes, and so may choose to breastfeed to normalise their experience [2]. The UK Confidential Enquiry into Maternal and Child Health (CEMACH) report, Pregnancy in women with type 1 and type 2 diabetes [3]*,* found 53% of type 1 and 2 women with diabetes intended to breastfeed, which was lower than that for the general population. However Simmons et al. from their study in New Zealand [2] found more positive intention and initiation rates than those in the UK CEMACH report [3].

Women with diabetes may have difficulty in breastfeeding after birth as they may have delayed lactogenesis [4], are more likely to have had assisted deliveries and their babies have a higher rate of admissions to the special care baby unit (SCBU). In order to have a supply of expressed colostrum ready for the first few days, women with diabetes are sometimes advised to hand express breast milk antenatally from 36 weeks gestation [5]. This should help with initiation of breastfeeding and the avoidance of the use of formula feeds or intravenous glucose.

Chapman et al. in their critical review of literature highlighted the scarcity of evidence on the safety and efficacy of antenatal breast expression, despite it being extensively encouraged via policies and commentaries [6]. An Australian study by Forster et al. reported a pilot study investigating the feasibility of developing a larger randomised controlled trial on the antenatal breast milk expression and the reduction in use of formula milk and found that: 40% of the infants of mothers who expressed antenatally received formula milk within the first 24 hours compared to 56% of infants of women with diabetes in pregnancy who did not express; the babies from the intervention group received less formula milk during their hospital stay; and they were more likely to be exclusively breastfed when leaving hospital (60% vs 44%) [7]. They also reported that 30% of the infants of mothers who expressed antenatally were admitted to SCBU compared to 17% of infants from the control group [7].

Our local guidelines stated that all women with diabetes should be advised to express their breast milk at 36 weeks gestation [8]. It is suggested that antenatal breast milk expression can alter the timing of birth, possibly through the breast stimulation, leading to the release of oxytocin acting on the uterus, with babies being born before 40 weeks [9], but this may be a positive result for women with diabetes past 37 weeks gestation. The Cochrane Review of breast stimulation for cervical ripening and induction of labour concluded that breast stimulation appeared to be beneficial in inducing labour and reducing postpartum haemorrhage, but may not be safe in high risk populations [9].

This report presents findings on the antenatal breast expression advice and uptake, and its relationship with neonatal outcomes and gestational age at birth in women with diabetes. This data was collected as part of a retrospective cohort study of women with diabetes which explored breastfeeding practices and views among these women and factors associated with breastfeeding up to six months postpartum [10,11].

## Methods

A retrospective cohort study design was chosen, using the maternal clinical records already routinely collected and a self-completed questionnaire. The average number of births per year locally was 4,300. With an annual rate of 3% pregnancies with diabetes and assuming a 50% response rate [12], we considered a two year period for data collection to be adequate. All women with diabetes who participated in the study attended maternity services at Derby Hospitals NHS Foundation Trust, during a two year period from 2001 to 2003. Women with multiple pregnancies, with learning difficulties and those who had babies with congenital abnormalities were excluded. Out of 257 women with diabetes who attended our local maternity services within the study period, 235 were eligible for inclusion and were invited to the study.

Standard procedure was to follow the Ten Steps to Successful Breastfeeding [13,14], and for women with diabetes to receive extra support and advice on blood sugar regulation, infant hypoglycaemic control policy and antenatal breast milk expression. The information provided explained the benefits of antenatal breast milk expression; for example having a supply of colostrum ready for the first few hours after birth when the baby may be hypoglycaemic, and that breastfeeding reduces the risk of type 1 diabetes; the best time to perform antenatal breast milk expression (36 weeks) and the practicalities of hand expressing and storing in sterile containers [8].

The questionnaire was developed from the routine data collection audit tool used by the maternity services up to discharge from the hospital ward. This was adapted to capture additional information about feeding up to six months and questions related to our objectives [10].

The questionnaire covered demographic characteristics, infant feeding practice up to six months, birth details including gestational age and weight, type of diabetes and glucose levels during pregnancy, recall of midwife advice regarding antenatal breast milk expression, monitoring the baby's glucose levels and breastfeeding. Participants were asked to indicate if they were advised to express breast milk antenatally and if so, at what time did they start, with this question providing the results for this report.

Ethical approval was obtained from the Local Research Ethics Committee (REC reference number: 0401/846). Women were sent a full information sheet, a questionnaire and a prepaid envelope to respond. No explicit consent was sought as the questionnaires were for self-completion and return, with inherent implied consent.

The average time postpartum that participants received their questionnaire was 20 months (SD = 7, range 9 to 32).

Data analysis: Mann–Whitney U tests were used to compare continuous variables and Chi-square tests were used for the categorical data. The mean difference and the difference in the proportions with the 95% confidence intervals (CI) are provided for primary clinical outcomes (gestational age at birth and admission to SCBU).

## Results

From the 235 women who were invited to the study, 94 (40%) responded to take part, of which 15 had type 1 diabetes mellitus, 11 had type 2 diabetes mellitus, and 68 had gestational diabetes. In total, 17% (16/94) of women in this study stated that they had expressed their breast milk antenatally. Thirty seven percent of women (35/94) reported that they were advised to express breast milk antenatally, of which 46% (16/35) commenced to do so at a mean gestational age of 36 weeks (SD: 1.76, range: 33 to 38).

Table 1 presents early pregnancy maternal characteristics which were collected from antenatal records. There were no significant differences between the groups in maternal age, gravida and body mass index (weight (kg)/height^2^ (cm)). There were no significant differences between the two groups of women who did not and those who did express their breast milk in the mean age at which they had left education (Mean (SD): 18.2 (3.3) vs 19.3 (2.5) p = 0.60 respectively). However, looking at proportions, while overall 17% of women expressed breast milk antenatally, in women who completed their education by 19 years, only (8/60) 13% expressed, compared to (8/21) 38% of women who had a higher level of education.

**Table 1:** Maternal characteristics

		**Expressed antenatally**	**Did not express antenatally**	**p value**
		**N=16**	**N= 69**	
		**Mean (SD)**	**Mean (SD)**	
**Age**		34.6 (4.2)	34.5 (4.7)	0.9
**BMI**		28.2 (7.4)	26.8 (6.5)	0.5
		n/N (%)	n/N (%)	
**Age completed education**	15-19 years	8/60 (13)	52/60 (87)	0.2
	21-23 years	8/21 (38)	13/21 (62)	
	25-30 years	0/4 (0)	4/4 (100)	
**Gravida**	G1	5/33 (15)	28/33 (85)	0.1
	G2	6/32 (19)	26/32 (81)	
	G3 or more	5/20 (25)	15/20 (75)	

Table 2 shows there were no significant differences between the antenatal breast milk expressing and non-expressing women in their infants’ Apgar scores, birth weight and length of breastfeeding. A reduction was observed in their gestational age at birth (Mean (SD): 37.1 (2.6) vs 38.2 (2.2), p = 0.06) for those who did antenatal expression compared to those who did not. The mean difference and 95% CI between the two groups were −1.2 (−2.4 to 0.04).

**Table 2:** Comparison between those who expressed antenatally and those who did not express against key indicators

	**Expressed antenatally**	**Did not express antenatally**	**p value**
	**N=16**	**N= 69**	
	**Mean (SD)**	**Mean (SD)**	
**Induction of labour**	7/15 (47)	37/67 (55)	0.57
**Vaginal birth**	4/16 (25)	28/70 (40)	0.23
**Gestational age at birth (wks)**	37.1 (2.6)	38.2 (2.2)	0.06
**Birth weight (g)**	3157 (770)	3279 (590)	0.95
**Apgar (5 minutes)**	8.4 (1.1)	8.3 (1.6)	0.88
**Breastfeeding duration (wks)**	18.5 (14.4)	19.9 (21.4)	0.19
	n/N (%)*	n/N (%)	
**SCBU admission**	5/15 (33)	8/66 (12)	0.06
Breastfed at birth**	15/15 (100)	60/70 (86)	0.19
Breast problems***	5/16 (31)	39/70 (56)	0.10

*: Where the numbers do not add up is because of the missing data (some questions were not answered by the participants).

**: The first feed was breast milk.

***Breast problems included complications such as cracked nipples and mastitis.

More babies (33%) from the antenatal expression group were admitted to the SCBU compared to the non-expressed group (12%) (p = 0.06). The difference in the proportion of babies who were admitted to SCBU between the two groups was 21% (95% CI: -3.9 to 46.3).

The overall initiation rate of breastfeeding in this small study was 88%. All the women in the antenatal expression group breastfed for their first feed versus 86% of those who did not undertake antenatal breast expression, although the difference between groups was not statistically significant (p = 0.19). There were no significant differences between the two groups with regard to the proportion of women who had induced labour (47% vs 55%), vaginal birth (25% vs 40%) or breast problems (31% vs 55%).

## Discussion

Evidence on antenatal breast milk expression is very limited as few studies [5,7,15] have ever been published on this topic. In our study, less than half of those women who recalled being advised to express breast milk complied with this recommendation and reasons for this need to be explored.

The findings on gestational age and admission to SCBU were clinically important as there were indications of differences between the two groups. Considering 95% confidence intervals, we would need a larger sample size to estimate the true differences with more precision. Our results of slightly lower gestational age were in line with the findings from Kavanagh et al., which showed a link between breast stimulation and induction of labour [9]. Forster et al. also found that more babies from the breast expression group were admitted to SCBU [7]. They hypothesised that this finding could be by chance or could be due to the mothers’ reluctance to allow formula feeding, as the majority of admissions were due to hypoglycaemia. It may also be due to a lower gestational age at birth, as indicated in our sample, induced by breast stimulation in mothers who had expressed antenatally.

All the women in the antenatal expression group breastfed as their first feed, with perhaps the antenatal expression giving those women confidence and practice so they were psychologically and physically prepared to breastfeed after the birth. However, it could be simply because of their higher rate of motivation and interest in breastfeeding. Eighty-five percent of women with diabetes who did not express antenatally also initiated breastfeeding. This, in line with findings from Simmons et al. [2], is of importance, as it may indicate a high level of commitment in initiating breastfeeding among women with diabetes in spite of the possibility of having delayed lactogenesis [4]. This is particularly worthy of notice as the breastfeeding initiation rate in local women without diabetes was 71% at birth [10].

There is a strong association between obesity and gestational diabetes, with obese women less likely to initiate breastfeeding and more likely to stop early [16]. Lower socio-economic status has also been shown to be an important predictor of lower breastfeeding rates in the general population [12] and in women with diabetes [11]. Although a larger proportion of women with a higher level of education (38%) expressed antenatally compared to 13% of women who left education by 19 years, the differences among the levels of education were not statistically significant between the two groups (Table 1). The lack of significant differences (as an indicator of socio-economic status) may be due to the limitations with the sample size.

The trends identified in our study, although small in sample size, can inform the design of larger projects and raise attention to the importance of confounding factors such as co-morbidities and psychosocial factors.

### Limitations

The reasons why women chose to follow or ignore the advice to perform antenatal hand breast milk expression were not explored in this study. Further in-depth research on communicating such messages and the underlying decision-making processes by women for antenatal breastfeeding expression, with a particular focus on issues related to acceptability of the practice, women's concerns and cultural background, would be beneficial.

This study was retrospective and self-reported questionnaires are prone to selective recall bias, although information such as birth weight, gestational age and specific complications were checked in maternal records which took precedent over recall. The average time postpartum that participants received their questionnaire was 20 months, which was a considerable time to remember details and memories could be confused with any subsequent pregnancies. However, experiences in pregnancy, birth and the postpartum period are significant events in a woman's life, so accurate recall is likely to be fairly high [17].

The sample size was fairly small, and from one hospital in the UK, so may not be the same in other parts of the country, or with other populations of women who may have different attitudes and experiences of antenatal breast milk expression. The small sample size may also have contributed to the lack of statistically significant differences between the groups. The study response rate was 40% and so it could be presumed that those most engaged would have returned their questionnaires and taken part, however the general characteristics of the subjects were similar to those from national data [10].

## Conclusions

More than half of the women who recalled being advised to undertake antenatal expression of breast milk, did not do so. There seems to be a trend between antenatal breast milk expression and lower gestational age at birth. The trend of a higher rate of SCBU admission for babies from the breast milk expression group compared to those who did not express antenatally, is of concern.

Further work is needed to explore the medical benefits and potential risks of antenatal breast milk expression and lower gestational age. A larger study is needed to determine whether the babies born to mothers who expressed antenatally are at an increased risk of SCBU admission, or whether our results showing a slight increase in admissions was just a coincidence and due to small sample sizes. Work is needed to explore women's feelings around the issue and their acceptability of the practice, whilst being aware that family members and partner's views and feelings about antenatal breast milk expression may influence a woman's decision to try this method. Work could also explore health professionals' views, training and current practice in relation to antenatal breast expression.

## Abbreviations

GDM: Gestational diabetes mellitus; SCBU: Special care baby unit.

## Competing interests

The authors declare that they have no competing interests.

## Authors’ contributions

HS led the study design, data collection, analysis, interpretation and critically revised and finalised the manuscript. AS drafted the original manuscript from an initial presentation by HS. Both authors read and approved the final manuscript.
